# The effects of yoga-based interventions on postnatal mental health and well-being: A systematic review

**DOI:** 10.1016/j.heliyon.2024.e25455

**Published:** 2024-01-29

**Authors:** Lydia Munns, Nicola Spark, Anna Crossland, Catherine Preston

**Affiliations:** a Department of Psychology, University of York, York, United Kingdom; b York and Scarborough Teaching Hospitals NHS Foundation Trust, York, United Kingdom

**Keywords:** Postnatal, Wellbeing, Mental health, Yoga

## Abstract

**Background:**

The postnatal period is a critical time for maternal mental health, presenting unique challenges and vulnerabilities. Identifying effective and accessible strategies to improve postnatal mental health and well-being is therefore crucial and could have substantial benefits for both mothers and babies, alongside broader implications for healthcare systems. Yoga is a potential intervention that has demonstrated notable benefits; however, a gap exists in systematically evaluating the existing literature on postnatal yoga-based interventions. This systematic review addresses this, aiming to comprehensively assess the impact of postnatal yoga on maternal mental health and well-being.

**Methods:**

Six databases were searched using keywords “yoga”, “yogic”, “postnatal”, “postpartum”, “perinatal”, “maternal”, “mother*“. Articles were considered if they were quantitative and evaluated a yoga or yoga-based intervention in postnatal samples. Study outcomes were extracted and synthesised descriptively. A quality assessment of studies was also conducted.

**Findings:**

Of the 383 non-duplicated records that were identified, nine met criteria for full-text review. Only 6 met the inclusion criteria and so were included in the review. Across the 6 studies within this review, data from 377 adult women were included and looked at the outcomes of women in the USA, Northern Ireland, Taiwan and Turkey. The findings of the studies suggest that taking part in postnatal yoga is associated with decreased symptoms of depression, an increase in psychological well-being and quality of life.

**Key conclusions:**

Yoga-based interventions may offer a promising and effective intervention for maternal mental health and well-being. However, due to the limited number of studies, and a lack of consistency in study design and measures, more high-quality research is required to establish these effects and explore the potential benefits on other aspects of maternal well-being and infant outcomes.

## Highlights

• There is a scarcity of research around postnatal yoga interventions.
• Postnatal yoga is associated with a decrease in mother’s depressive symptoms.
• Postnatal yoga has been found to increase mother’s psychological well-being and quality of life.
• New mothers are willing to engage in postnatal yoga as an intervention.

## 1 Introduction

The postnatal period is linked to vulnerabilities in mental health with substantial social and economic consequences. Postnatal depression is thought to affect between 6.5 % and 20 % of women within a year of giving birth [1] and perinatal mental health issues are estimated to cost the UK £8.1 billion per annum [2]. Poor postnatal mental health and wellbeing is linked to problems with physical health and recovery, including postpartum weight retention, pain and physical functioning [3]. There is a known bidirectional relationship between physical and mental health among postpartum women, suggesting the importance of concurrently targeting both physical and mental health to promote perinatal health [4]. Furthermore, mental health difficulties in the mother/birthing parent can also negatively impact cognitive and psychosocial developmental and psychological outcomes for the infant such as anxiety, depression and externalising problems [5,6]. Thus, effective and deliverable, strategies for improving postnatal mental health and well-being could have substantial benefits to mothers, babies, the healthcare system, and wider society.

One such strategy is yoga, a form of mind-body exercise combining physical posture, meditation, and breathing techniques [7]. The popularity of yoga as part of healthcare in western culture is increasing [8] and so the need to effectively assess its efficacy is becoming more important. There have been recent systematic reviews and meta-analyses assessing research on the efficacy of yoga during the prenatal period [[9], [10], [11], [12]]. These reviews, focusing only on the prenatal period, have found yoga to have positive impacts on mood, stress, pain and social relationships, showing support for yoga during pregnancy as a promising non-pharmacological intervention to improve mental health and well-being [9,11,12]. However, no reviews have been done focusing on the benefits of yoga solely postnatally, which is concerning given the fact that historically, pregnancy has been suggested as protective against depression, with the postpartum period carrying an increased risk for developing major depressive disorder [13]. Although this theory has been called into question [14], current postnatal depression rates continue to highlight the importance of research examining postnatal interventions.

The potential benefits of postnatal yoga-based interventions could act through multiple mechanisms. For example, yoga can help regulate the hypothalamic-pituitary-adrenal (HPA) axis that plays a role in how people respond to stressors [15]. The review by Chen and colleagues (2017) found four studies across multiple countries that revealed consistently lower salivary cortisol levels in pregnant women after they had regularly practiced yoga. These findings strongly suggest a positive impact of yoga on reducing stress levels, which can lead to better mood and reduced anxiety. It is also thought that yoga can improve aspects of physical health, with a recent review identifying multiple studies that reported pregnancy yoga increased women’s pain tolerance [16], highlighting the potential for postnatal yoga to result in similar benefits. Finally, yoga has been linked with the bond between a mother and her unborn baby. A recent paper found that women who completed an 8-week pregnancy yoga course scored significantly higher on measures of antenatal attachment compared to controls [17]. Neurobiological mechanisms behind this link have been suggested, with yoga being found to enhance levels of oxytocin in psychiatric samples (a hormone involved in emotional and social bonding) [18]. However, this potential mechanism has not yet been investigated in the context of the perinatal period.

To date, assessment of the literature surrounding studies on yoga interventions during the postnatal period is lacking. Although reviews have looked generally at nonpharmacological interventions to improve wellbeing during the perinatal period [19,20], none focus solely on yoga, and or consider the postnatal period in isolation. Nonpharmacological treatments are particularly preferable for addressing postnatal mental health difficulties due to their potential for longer term benefits and fewer side effects [21]. By honing in on yoga interventions specifically, we can investigate the distinct benefits it may offer in promoting postnatal wellbeing, particularly because the antenatal and postnatal periods are significantly different. Therefore, this review examines quantitative studies that assess yoga-based interventions in postnatal samples.

## 2 Methods

This review was registered in Prospero (ID: CRD42022366873) prospectively, before data extraction. The methodology of this systematic review follows the Joanna Briggs Institute Methodology for Effectiveness Reviews [22]. A systematic review specialist was consulted for general advice around conducting a systematic search. Literature searches were conducted to identify all published articles on interventions involving yoga during the postnatal period. A meta-analysis was considered if enough papers (2+) incorporating equivalent outcome, measures, population sample and interventions were identified.

### 2.1 Search strategy

The databases searched included: Scopus, PubMed, Web of Science, PsychInfo, Medline & Embase. These electronic databases were searched in April 2022. Grey literature was not searched to ensure all studies were peer-reviewed. Our search strategy was the following across all databases: TITLE-ABS-KEY (postnatal OR postpartum OR perinatal OR maternal OR mother*) AND (Yoga OR Yogic). English language and human research limits were set. See Table 1 in the supplementary material to see further information on search strategies for each database. All searches were uploaded to Rayyan (www.rayyan.ai; [23]) through which titles and abstracts were examined for potentially relevant articles.

**Fig. 1:**
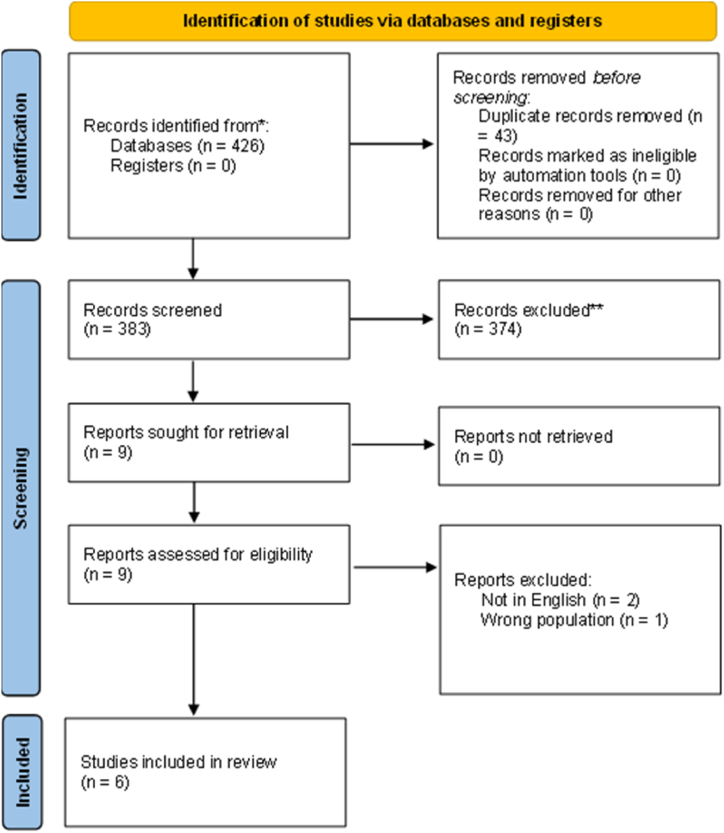
A PRISMA flow diagram of the systematic screening process, in accordance with PRISMA 2020 guidelines [24].

See Fig. 1 for the PRISMA diagram of the screening process, in accordance with the PRISMA 2020 statement [24].

### 2.2 Inclusion/exclusion criteria

This review aimed to identify any quantitative study that investigated the relationship between yoga-based interventions and maternal postnatal mental health and wellbeing. Only experimental designs were considered, including either a comparison/control group, or comparisons between outcome measures pre and post intervention. No specific outcomes were identified before the systematic search and any studies that considered outcomes related to maternal postnatal mental health and wellbeing were considered. Only postnatal outcomes were considered in research where the whole perinatal period was studied and only quantitative studies, which directly tested an intervention that included yoga activities for the mother and/or infant in a postnatal sample were included. For the purpose of this review, the postnatal period was considered as up to 12 months following birth, given that recent literature suggests women should receive postpartum care for up to 1 year following the birth of their child [25]. The independent variable had to be a yoga-based intervention for mother and/or baby and the dependent variable had to be postnatal mental health and well-being. Papers not written in the English language were not included to ensure that the review was feasible given the available resources, time constraints and the fact that all authors were English speaking. Refer to Table 2 in the supplementary material for information on the inclusion and exclusion criteria.

### 2.3 Quality assessment

The quality of studies was assessed by two reviewers using the Joanna Briggs Institute Critical Appraisal Checklist for Quasi-Experimental Studies [22] or Randomised-Controlled Trials [26] depending on the design of the study.

## 3 Results

Six papers were included in this review. A meta-analysis wasn’t possible due to the limited number of papers identified and the variability in outcome measures, samples and interventions between papers. Three studies specifically measured depression but varied in sample characteristics—one used a community sample [27], another focused on high depression scores [28], and the third included individuals throughout the perinatal period without separating postnatal data [29]. This variation hinders a meta-analysis. Similarly, two studies that assessed quality of life studies using the same measure (SF-36) had differing populations—one from a community sample [30] and the other exclusively with high depression scores [28], making meta-analysis unsuitable.

See Table 3 for further study information.

**Table 3:** Summaries of studies.

Author & date	Title	Sample size and population	Design	Intervention	Outcome measures	Results/conclusions
**Timlin & Simpson, 2017**	A preliminary randomised control trial of the effects of Dru yoga on psychological well-being in Northern Irish first-time mothers.	n = 32 (16 = yoga, 16 = WLC^a^). Postnatal participants between 6 weeks and 1 year following birth. All participants were first-time mothers. Range 8–50 weeks postpartum.	RCT^b^	4 weeks of Dru yoga (Hatha).	Perceived Stress scale; Positive and Negative Affect Scale (PANAS); Brief COPE inventory.	- Significant time by group interactions were found. - Compared to the control group, the yoga group shows reduced stress (*p* = 0.001), negative affect (*p* = 0.001) & dysfunctional coping (*p* = 0.042) and an increase in problem focused coping (*p* = 0.001).
**Buttner et al. 2015**	Efficacy of yoga for depressed postpartum women: A randomised controlled trial.	n = 57 (28 = yoga, 29 = WLC). Postnatal participants between 6 weeks and 1 year following birth and scoring over 12 on HDRS (depression).	RCT	8 weeks (16 sessions) of vinyasa (Ashtanga) yoga. Same sequence in each session.	The Hamilton Depression Rating Scale (HDRS); The Inventory of Depression and Anxiety Symptoms (IDAS); The Medical Outcomes Study 36-Item Short-Form Health Survey (SF-36).	- Both groups showed improvements (*p* < 0.001). - The yoga group demonstrated significantly greater improvement on all measures (HDRS: p = 0.005, IDAS Depression: *p* = 0.002, IDAS Anxiety: *p* = 0.006, IDAS Trauma: *p* = 0.021, SF-36: *p* < 0.001).
**Ko et al., 2012**	Community-based postpartum exercise program.	n = 23 (28 without attrition). Postnatal participants between 2- and 6-months following birth.	Quasi- experiment Within-subjects	Once a week for three months. Yoga & Pilates exercise, stretching, breathing, muscle contractions and low-intensity aerobics.	Centre for Epidemiological Studies Depression Scale (CES-D); Fatigue Symptoms Checklist (FSC); Body composition analyser	- Participants with high depression scores at baseline demonstrated a significant reduction in depression symptoms following the intervention (*p* = 0.021). Low scorers did not. - No significant effects on fatigue, or BMI. Weight (*p* < 0.001) and body fat (*p* < 0.001) significantly reduced, and fat loss significantly increased (*p* < 0.001).
**Miklowitz et al. 2015**	Mindfulness-Based Cognitive Therapy for Perinatal Women with Depression or Bipolar Spectrum Disorder.	n = 39 (attended at least one session). Pregnant, up to one year postnatal (up to 1 year after birth), non-pregnant but trying to conceive. Diagnosis of major depression or bipolar disorder, current subthreshold depressive symptoms.	Quasi- experiment Within-subjects	8 weekly sessions of mindfulness training (including yoga).	Beck Depression Inventory (BDI-II), The Hamilton Depression Rating Scale (HDRS); Young Mania Rating Scale (YMRS); State-Trait Anxiety Inventory-Current Status Scale (STAI-C); Longitudinal Interval Follow-up Evaluation (LIFE); Psychiatric Status Ratings (PSR); Five Facet Mindfulness Questionnaire (FFMQ).	- Depression decreased across the entire sample (*p* = 0.008) - No difference was found between perinatal stage (*p* = 0.43). Authors did not single out results for postpartum participants. - Those who had increases in mindful tendencies showed decreases in depression (*p* = 0.048). - There were no significant differences in anxiety scores between groups.
Cameron & Shepherd 2018*	Evaluation of outcomes from an evidence-based programme for mothers and babies.	n = 66 (33 = intervention, 33 = control) postnatal participants 0–3 months following birth.	Quasi- experiment Between-subjects	8 weekly sessions of baby yoga (4 weeks) or baby massage (4 weeks) songs rhyme and discussion topic.	Dyadic synchrony (Infant CARE-Index (ICI) - video interaction) questionnaires - My Baby scale; Mother to Infant Bonding scale; Warwick-Edinburgh Mental Well-being Scale, qualitative responses.	- No significant differences between intervention and control group. - Positive qualitative experiences of participants led to continuation of the sessions.
**Unver & Timur Tashan 2021**	Effect of yoga on posttraumatic growth and quality of life in first-time mothers: A randomised controlled trial.	N = 160 (80 = intervention, 80 = control) postnatal participants 2–6 months following birth.	RCT	10 weekly sessions of yoga. Same sequence in each session.	Posttraumatic Growth Inventory (PTGI); Short Form 36 Item quality of life questionnaire (SF-36).	- Significant differences between intervention and control group for both post traumatic growth (*p* < 0.001) and quality of life (*p* < 0.001).

*No *p* values provided.

a WLC = Wait list control.

b RCT = Randomised controlled trial.

### 3.1 Description of studies

#### 3.1.1 Date and location

The studies identified were published between 2012 and 2021. Two of the studies were conducted in the USA [28,29], two in Northern Ireland [31,32] one in Taiwan [27] and one in Turkey [30].

#### 3.1.2 Participants and attrition

One study was conducted on individuals with a clinical diagnosis for major or bipolar depression [29] and one study was conducted on a sample with current depressive symptoms [28]. The other studies were conducted on community samples [27,[30], [31], [32]]. One study included participants throughout the perinatal period, from conception to post-birth [29]. All other studies were conducted on exclusively postnatal samples.

All studies reported a relatively low attrition rate when excluding individuals who did not meet inclusion criteria and did not give consent ( ≤ 20 %). One study required participants to attend at least one session for their data to be included [29].

#### 3.1.3 Intervention

One study included a yoga intervention aimed only at the infant, where yoga was only delivered in half of the eight sessions [31]. All other studies included interventions aimed exclusively at the mother/birthing parent. The type of yoga was specified in three of the studies: Dru (Hatha) [32], Vinyasa (Ashtanga) [28], and Mindful-yoga [29]. The other studies did not specify. The duration of the yoga-based intervention varied, from 4 weeks [31,32] and 8 weeks [28,29], to 10 weeks [30] and 12 weeks [27]. Three studies used yoga only in conjunction with other exercises/activities [27,29,31] and two studies also provided participants with a DVD to practice yoga at home [28,32], although this was only taken up by a minority of participants.

Half of the studies included mothers that were between six weeks to one year following the birth [28,29,32], with the other half selecting participants within a shorter postpartum period, of between two and six months [27,30] and zero and three months [31] following birth.

Four of the studies had control groups [28,[30], [31], [32]], which were treated as usual (no yoga) or waiting list controls (waiting for yoga intervention).

#### 3.1.4 Measurement tools

Measures assessing depression, physiological changes, the mother-infant relationship, and quality of life were identified among the 6 studies.

Depression was measured by 3 of the studies [[27], [28], [29]], using a different assessments, including The Hamilton Depression Rating Scale (HDRS), The Beck Depression Inventory (BDI-II), The Inventory of Depression and Anxiety Symptoms (IDAS), and The Centre for Epidemiological Studies Depression Scale (CES-D), with a 4th study [32] assessing mood more generally using the Positive and Negative Affect Scale (PANAS).

Physiological changes were assessed by one study [27], using The Fatigue Symptoms Checklist (FSC) and a body composition analyser. The mother-infant relationship was similarly only assessed by one study [31] and measured using the Infant CARE-Index (ICI) and the Mother to Infant Bonding scale. Quality of life was assessed by two studies [28,30] using the same measurement, the study 36-Item Short-Form Health Survey (SF-36).

Psychological well-being was also measured in many of the studies using different validated measurement tool. For details see Table 3.

## 4 Effects of the interventions

### 4.1 Depression

All but two studies [30,31] measured maternal depression [[27], [28], [29]] or mood [32] as a primary outcome variable.

Statistical analysis varied between studies. Comparisons to a control group revealed that yoga practice demonstrated a decline in negative affect (*p* = 0.001) [32] and a steeper linear decline in depressive symptoms over the course of the intervention compared to controls (*p* < 0.001) [28]. In the one-sample studies comparing pre and post-intervention measures, there were significant reductions in depressive symptoms for individuals with diagnoses of major or bipolar depression (*p* = 0.008) [29] or in non-clinical samples with high-levels of depressive symptoms at baseline (*p* = 0.021) [27]. In the clinical sample [29] mean scores of depression at pre and post-intervention reflect participants moving from a mildly to a minimally depressed state.

The same study also examined depressive symptoms at one and six months post intervention and found only seven participants from the 32 who had follow-up data at six months met criteria for major depression, none met criteria for mania or mixed disorder [29]. However, there was no control group for comparison and this study didn’t separate their results by perinatal stage (pre, during and post pregnancy). Despite this, no significant differences were found in the reduction of depression scores between perinatal stage.

### 4.2 Maternal psychological well-being

All studies included other measures of maternal psychological well-being, although all utilising different measures. When compared to a wait-list control group it was found that the intervention group demonstrated a greater reduction in stress, negative affect and dysfunctional coping, and an increase in problem focused coping (*p* = 0.001) [32] as well as a steeper decline in traumatic intrusions (*p* = 0.021) and social anxiety (*p* = 0.006) [28]. Post traumatic growth, which is defined as transformations that occur following a traumatic experience (i.e. birth trauma), was assessed in one study, which found that this was higher in the yoga group compared to the control group (*p* < 0.001) [30]. This was also the case for the post-traumatic growth inventory subscales of: change in self-perception, the philosophy of life and relationships with others (all *p* values < 0.001).

One study, whose intervention was based on mindfulness and included mindful yoga [29] found that mindfulness tendencies increased from pre-to post intervention. Furthermore, the same study also found that those who had greater increases in mindfulness tendencies also demonstrated greater reductions in depressive symptoms (*p* = 0.048).

No significant effects were found for feelings of panic [28], fatigue [27], general anxiety or mania symptoms [29] and no improvements in mental well-being (assessing feelings of self-acceptance and positive affect) were found with baby yoga [31].

### 4.3 Physiological changes

One study [27] measured changes in body composition including weight, BMI, body fat, fat-free mass and total body water. Comparing pre- and post-intervention measures they found a significant reduction in weight (*p* < 0.001), fat mass (*p* < 0.001) and fat loss (*p* < 0.001), but not BMI, fat-free mass or total body water. However, this study did not have a control group for comparison.

Mother-infant relationship.

One study [31] took measures concerning the mother/birthing parent-infant relationship. This included videoed interactions which were coded for dyadic synchrony as well as measures of subjective feelings about the mothers’ bond with their baby. None of these measures revealed any significant effects compared to the control group.

### 4.4 Quality of life

Two studies [28,30] gathered quality of life data, which involved asking questions about general, physical, and mental health. The first study found that those who participated in yoga had a significantly steeper linear increase in quality-of-life scores (*p* < 0.001) during the intervention, compared to waiting list controls [28]. The second study used the same assessment to measure quality of life (short form-36 item health related quality of life scale) but looked in more detail at the subscales to find that issues with physical functioning, bodily pain, general health, social functioning, and vitality were all significantly lower in the yoga group compared to the control group (all *p* values < 0.001) [30].

### 4.5 Satisfaction with the interventions

One study [32] conducted focus groups to assess the subjective experience of taking part in the intervention. Content analysis was used to determine the main themes. Overall, the intervention was positively received. Facilitators to attend the classes were identified and included convenience (classes being at a convenient time and location), having someone to look after the baby and an awareness of the health benefits of yoga. Barriers to attending were identified as tiredness, not being comfortable with their postnatal body shape, and an instructor that they felt did not understand their needs.

Another study [29] measured satisfaction with the course using the Client Satisfaction Questionnaire. All participants who completed the programme rated high levels of satisfaction.

### 4.6 Quality assessment

According to the JBI Critical Appraisal Checklist for Quasi-Experimental Studies, two studies received a score of 5/7 [27,29] and the final study received a score of 7/9 [31]. The two former studies did not include a control group meaning that there was no comparison of the treated group with a group receiving no treatment, so any causal plausibility is weaker. See the full quality assessment in Table 4 of the supplementary materials.

According to the JBI Critical Appraisal Checklist for RCT’s, studies received a score of 7/11 [30], 8/11 [32] and 10/11 [28]. As all 3 RCT studies included no treatment or wait list controls, there was no blinding for participants and instructors. There was also no information as to whether the individuals conducting the analysis were blinded, suggesting potential bias related to administration of the intervention. For the study with the lowest quality score, the method of participant allocation was suboptimal, as this was based on which family health centre they were from (3 family health centres were randomly allocated to each group) [30]. See the full quality assessment in Table 5 of the supplementary materials.

None of the 6 studies had a complete follow up, and differences between groups in terms of their follow up were not adequately described and analysed which could represent a threat to internal validity.

## 5 Discussion

The review has collated studies investigating the effect of yoga-based interventions on postnatal maternal outcomes and has uncovered three key findings. Firstly, studies examining the potential benefits of postnatal yoga are scarce. Only six studies were found following a systematic search of the literature and these varied in terms of type of intervention, study design, analysis and target sample. All but one study only delivered the intervention to the mother/birthing parent, with the remaining study requiring the parent to deliver the intervention to the baby [31]. No studies to date examine the potential benefits of mother/parent *and* baby yoga and there is little consistency between studies as to what is considered the ‘postnatal period’, with participants ranging from 0 to 12 months post birth.

The second key finding is that yoga-based interventions may have a positive impact on symptoms of depression postnatally. All studies measuring depressive symptoms or low mood found a significant decrease in symptoms following the intervention, especially in those with higher-levels of depression at baseline. Further to this, quality of life, psychological growth and physical benefits were found to be improved following yoga, suggesting that interventions such as this may have wider psychological and physical benefits. These results support the positive outcomes associated with yoga-based interventions that have been found in antenatal literature [9,11,12], suggesting some consistency between perinatal periods.

The final key finding is that yoga-based interventions seem to be acceptable to the participants. All studies received positive feedback from those attending, and/or demonstrated low attrition rates. In addition, no adverse effects were reported, although most studies did not include additional follow ups to assess more longer-term consequences of taking part. This suggests that such interventions may be well received by the target audience and thus may offer a logistically feasible method to be implemented in the community.

From these main findings, the potential benefits and feasibility of postnatal yoga-based interventions are made clear. These findings and future research in this area has the potential to impact clinical health practices, health care policy and public awareness of effective and accessible postnatal nonpharmacological interventions. This is particularly important, given the potential for yoga-based interventions to be virtually accessible, and an opportunity for community building during a period of potential isolation for mothers [33]. It is hoped that this review, by providing a foundation of evidence for a nonpharmacological yoga-based postnatal interventions, will facilitate future research and intervention development to improve maternal wellbeing in the first 12 months following birth, without side effects associated with pharmacological treatments [21].

However, research in this area is scarce, with inconsistencies in measures taken, study design, target population, types of intervention and data analysis. All studies were found to have significant shortcomings, and this means that conclusions drawn based on the current literature need to be taken with caution. For example, one of the RCT studies had a suboptimal method of participant randomisation [30] and only one study considered weeks postpartum as a contributing factor to maternal outcomes in their analysis [32]. Most studies also did not require participants to refrain from other forms of yoga practice/postnatal groups or exercise. Sample sizes for most of the studies were small (ranging from 23 to 160). Despite some reporting a power analysis to justify the sample size, it is unclear what the estimated effect size was based on and whether such calculations were appropriate for all analyses that were conducted, including subgroup analysis [[28], [29], [30]]. Additionally, samples in all studies were self-selecting and most participants were middle class, which limits the generalisability of the research findings, especially when considering the diverse range of postnatal experiences influenced by factors such as ethnicity [34].

Outcomes measured were also inconsistent between studies, with only one study examining possible effects on the mother/parent-infant relationship [31] and one examining psychological growth [30]. The studies were also limited when thinking about longer-term outcomes. Only one study [29] included longer-term follow-ups but this study sample was not exclusively postnatal, consisted of participants with prior mental health diagnoses and did not include a control group for comparison. Therefore, although short-term effects might seem promising, longer-term outcomes of taking part in a postnatal yoga-based intervention are unclear. Further to this, only one study measured physiological outcomes pre- and post-intervention, which allows us to identify whether the yoga intervention is associated with significant physical change, however this study did not have a control group [27]. The impact of yoga on a mother’s physicality and physiology is an important area for future research, given the fact that yoga is primarily a physical activity, postnatal physical and psychological health are intrinsically linked [3], and the postnatal period involves profound physiological transformations [35]. Therefore, the known benefits yoga can bring in regard to muscle strength [16] and reducing stress via the HPA [15] could have important physiological implications. Finally, considering specific limitations of this review, we only considered studies that had been though full peer review in order to ensure quality of study design. Including grey literature within this review may have broadened the scope and depth of this review by allowing for the identification of further studies and therefore, the potential for more consistency between study designs and outcomes measured as well as avoiding potential publication bias. Furthermore, limiting the review to only publications available in the English language may have also limited our access to appropriate studies. Due to the diversity in the studies examined we were unable to conduct a meta-analysis, expanding our search to grey literature and non-English texts may have provided us with sufficient data to do this and thus add further weight to the current findings.

## 6 Future directions

It is clear from these findings that more high-quality research is required, with larger and empirically justified sample sizes and effectively randomised control samples. Future research should aim to include a wider demographic of participants to determine efficacy and acceptability of yoga-based interventions across samples of different backgrounds. Additionally, further post-intervention follow-up assessments are required to examine the longer-term effects of the intervention on a wider range of outcome measures, particularly relating to the infant given the links between maternal mental-health and infant well-being [5,6]. A better understanding of physiological outcomes, such as weight loss, could further assess the potential physical benefits of yoga and its links to psychological factors.

Developing interventions that involve both mother/birthing parent and baby is another avenue to explore. A key reason identified in the studies as attributing to attrition for the mother/birthing parent only interventions, was difficulty obtaining childcare [27,28,32]. A yoga intervention that incorporates both the parent and infant means that any childcare issues would be limited (with the exception of potential older siblings), thus making the intervention more accessible and lowering attrition rates further.

## 7 Conclusion

Although current research is both scarce and limited, there is evidence for potential benefits of postnatal yoga, particularly on postnatal mood (depressive symptoms). Overall, it seems that such interventions are well received by those who take part and may therefore offer a prospective method for prevention and treatment of low mood following birth. However, more high-quality research in this area is imperative in order to establish these effects by addressing significant limitations in the current research, assessing longer-term effects of yoga practice and exploring interventions aimed at both mother/birthing parent and baby.

## Ethical approval

Not applicable.

## Funding sources

Economic and 10.13039/501100000269Social Science Research Council (ESRC), UK Grant (Anna Crossland).

Elsie May Sykes Award (UK) (Nicola Spark).

## CRediT authorship contribution statement

**Lydia Munns:** Writing – review & editing, Writing – original draft, Visualization, Project administration, Methodology, Investigation, Formal analysis, Data curation. **Nicola Spark:** Writing – review & editing, Conceptualization. **Anna Crossland:** Writing – review & editing, Writing – original draft, Data curation. **Catherine Preston:** Writing – review & editing, Writing – original draft, Supervision, Project administration, Methodology, Investigation, Formal analysis, Data curation, Conceptualization.

## Declaration of competing interest

The authors declare that they have no known competing financial interests or personal relationships that could have appeared to influence the work reported in this paper.
