# Supplementary material for: The effects of yoga-based interventions on postnatal mental health and well-being: A systematic review
Source: Heliyon. 2024 Jan 29;10(3):e25455. doi: 10.1016/j.heliyon.2024.e25455 (PMC10845905; doi:10.1016/j.heliyon.2024.e25455)
Supplement: Multimedia component 1 [file mmc1.docx]

## Supplementary Materials

**Table 1.**Search strategies for each database.

|  | ***Search Strategy*** |
| --- | --- |
| ***Scopus*** | TITLE-ABS-KEY ( postnatal OR postpartum OR perinatal OR maternal OR mother* ) AND TITLE-ABS-KEY ( 'yoga' OR 'yogic' ) AND ( LIMIT-TO ( LANGUAGE, "English" ) ) |
| ***PubMed*** | ('yoga'[Title/Abstract] OR 'yogic'[Title/Abstract]) AND (postnatal[Title/Abstract] OR postpartum[Title/Abstract] OR perinatal[Title/Abstract] OR maternal[Title/Abstract] OR mother*[Title/Abstract]) |
| ***Web of Science*** | (TI=(postnatal OR postpartum OR perinatal OR maternal OR mother*)) AND TI=('yoga' OR 'yogic') OR (AB=(postnatal OR postpartum OR perinatal OR maternal OR mother*)) AND AB=('yoga' OR 'yogic') OR (AK=(postnatal OR postpartum OR perinatal OR maternal OR mother*)) AND AK=('yoga' OR 'yogic') |
| ***OVID:***   - ***PsycINFO*** - ***Medline*** - ***Embase*** | 1. ('yoga' or 'yogic').ab,kf,ti.  2.(postnatal or postpartum or perinatal or maternal or mother*).ab,kf,ti.  3.1 and 2  4.limit 3 to English language  5.limit 4 to human |

**Table 2.** Inclusion and exclusion criteria.

|  | ***Inclusion criteria*** | ***Exclusion criteria*** |
| --- | --- | --- |
| ***Types of studies*** | - Experimental studies (including RCT’s and quasi-experimental studies)  - Full-text primary research papers  - Published in English  - No constraints on publication date | - Observational studies  - Studies with only qualitative assessments  - Cross-sectional studies  - Systematic reviews  - Peer reviews  - Commentaries |
| ***Participants*** | - Over 18 years of age  - Postnatal women/ birthing parents (between 0- and 12-months following birth) | - Under the age of 18 |
| ***Intervention*** | - A postnatal ‘yoga-based’ intervention: A postnatal programme including yoga for mother/birthing parent and/or baby | N/A |
| ***Comparators*** | - Studies must compare outcomes in some way, either through comparing a control group to an intervention group, or by comparing outcome measures before and after the intervention | N/A |
| ***Outcome measures*** | - Measures concerning maternal postnatal mental health and well-being (e.g., depression, anxiety, stress, mother-infant interaction, quality of life) | N/A |

**Table 4.**Quality Assessment for Quasi-Experimental Studies

|  | Ko et al., 2012 | Miklowitz et al. 2015 | Cameron & Shepherd 2018 |
| --- | --- | --- | --- |
| Clear ‘cause’ and ‘effect’ | Yes | Yes | Yes |
| Participants similar across comparisons | N/A | N/A | Yes |
| Comparison participants receiving similar treatment | N/A | N/A | No |
| Control group | No | No | Yes |
| Multiple measurements | Yes | Yes | Yes |
| Complete follow up | No | No | No |
| Outcome comparisons measured in the same way | Yes | Yes | Yes |
| Reliable outcome measures | Yes | Yes | Yes |
| Appropriate statistical analysis | Yes | Yes | Yes |
| Quality Assessment Score | **5/7** | **5/7** | **7/9** |

**Table 5.**Quality Assessment for RCT’s.

|  | Buttner et al. 2015 | Timlin & Simpson, 2017 | Ulver & Timur Tashan 2021 |
| --- | --- | --- | --- |
| True randomisation used | Yes | Yes | No |
| Allocation of treatment groups concealed | Yes | Unclear | Unclear |
| Treatment groups similar at baseline | Yes | Yes | Yes |
| Participants blinded to treatment assignment | N/A | N/A | N/A |
| Those delivering treatment blind to treatment assignment | N/A | N/A | N/A |
| Treatment groups treated identically other than intervention | Yes | Yes | Yes |
| Outcome assessors blind to treatment assignment | Yes | No | Unclear |
| Outcome measured in the same way for treatment groups | Yes | Yes | Yes |
| Outcomes measured in a reliable way | Yes | Yes | Yes |
| Complete follow-up | No | No | No |
| Participants analysed in the groups to which they were randomised | Yes | Yes | Yes |
| Was appropriate statistical analysis used? | Yes | Yes | Yes |
| Appropriate trial design | Yes | Yes | Yes |
| Quality Assessment Score/11 | **10** | **8** | **7** |
